# The Curious *U*: Integrating Theories Linking Knowledge and Information-Seeking Behavior

**DOI:** 10.1162/OPMI.a.41

**Published:** 2025-10-17

**Authors:** Alexandr Ten, Pierre-Yves Oudeyer, Michiko Sakaki, Kou Murayama

**Affiliations:** Hector Research Institute of Education Sciences and Psychology, University of Tübingen, Tübingen, Germany; Flowers AI & CogSci Lab, Inria, University of Bordeaux, Talence, France; Research Institute, Kochi University of Technology, Kochi, Japan

**Keywords:** information seeking, intrinsic motivation, interest, confidence, uncertainty

## Abstract

Many empirical studies have found a curvilinear (inverted-*U*) relationship between knowledge and curiosity, such that curiosity is induced when stimuli are neither unknown nor too familiar. While various theoretical accounts have been proposed to explain this phenomenon, no clear link between them have been delineated. In this Perspective, we review seven psychological accounts of the inverted-*U* relationship between knowledge and curiosity (“the *U*”) and provide a coherent framework integrating them. According to this framework, the *U* emerges as a consequence of the imperative to pursue learning progress and thus maximize knowledge. We show that some theories of curiosity address this issue by explicitly stipulating knowledge maximization as the computational objective, and learning-progress maximization as an optimal means of achieving it (i.e., *normative* theories). Other theories focus on psychological mechanisms or factors that drive curiosity (i.e., *process* theories). We propose that these process-theoretic mechanisms could also work in a manner that maximizes learning by signaling situations in which some relevant prior knowledge exists, but is incomplete. The implications of this framework for future theoretical work on curiosity and its connections to related phenomena are discussed.

## INTRODUCTION

Knowledge and curiosity are inseparable. Curiosity is often referred to as “the desire to know”. There is also a commonsense intuition regarding the relationship between curiosity and knowledge. For example, it seems obvious that one cannot be curious about learning an already known fact. At the same time, one is unlikely to be curious about a fact that is completely detached from one’s existing knowledge. Curiosity seems to manifest only in the presence of some but incomplete knowledge.

This commonsense intuition about a nonmonotonic relationship between knowledge and curiosity has received robust empirical support. For instance, when reading general knowledge questions, curiosity peaks when the confidence in knowing the answer is intermediate (Baranes et al., 2015; Dubey & Griffiths, 2020; Kang et al., 2009; Litman et al., 2005; Lyew et al., 2023; Spitzer et al., 2024). Moreover, participants exhibit behavioral and aesthetic preferences for stimuli that are only somewhat predictable compared with entirely predictable and completely unpredictable stimuli (Brielmann & Dayan, 2022; Geana et al., 2016; Kidd et al., 2012; Singh & Manjaly, 2021; Walker, 1981).

**Figure 1.**
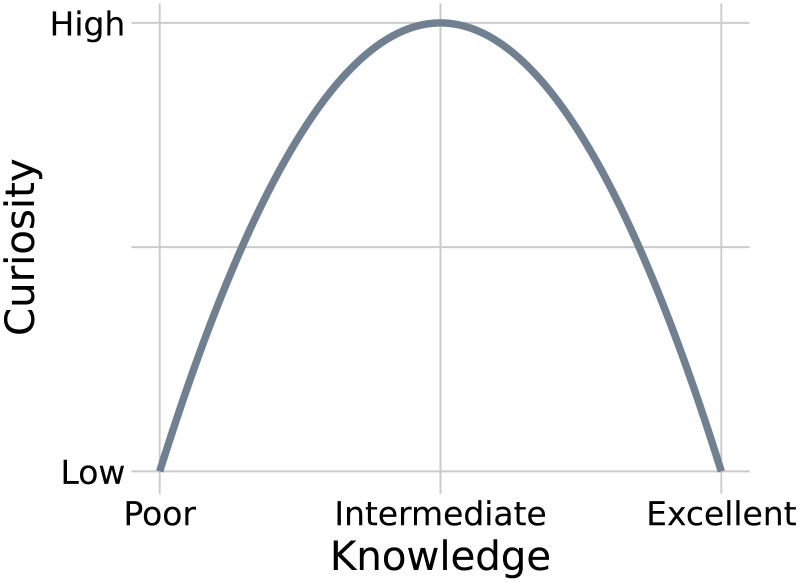
Inverted-*U* shaped relationship between knowledge and curiosity.

As knowledge is one of the main factors associated with curiosity, explaining their inverted-*U* relationship has been an important goal in several theories of curiosity (Litman, 2019; Silvia, 2006). However, despite its 80-year history, scientific accounts of this phenomenon have failed to converge on a single coherent explanation. One reason for this slow progress is that both curiosity and knowledge are unobservable constructs, which results in a diverse set of combinations of their operational definitions. For example, curiosity is often measured through various subjective ratings, choices, or various measures of the willingness to spend resources (e.g., time, money) to obtain information. Operationalizations of knowledge are even more diverse and may include various measures of confidence, competence, complexity, surprise, exposure time, etc. Here, we concern ourselves with theoretical explanations of the general inverted-*U* relationship between knowledge and curiosity, assumed to underlie diverse empirical demonstrations. We discuss seven prominent psychological theories that either predict or imply this relationship, which we refer to simply as “the *U*” (Figure 1), meaning, more precisely, a nonmonotonic, unimodal mapping from a function of knowledge to a function of curiosity.

Considering how different theories account for the *U* allows us to better understand how they are related and what they accomplish with respect to explaining curiosity. To foreshadow our conclusions, our synthesis portrays curiosity as arising from a set of adapted mechanisms that approximate optimal resource allocation for knowledge accumulation. More concisely, curiosity attempts to optimize knowledge under certain constraints, and thus the *U* emerges. In this view, maximization of knowledge is the fundamental principle underlying all reviewed theories, notwithstanding their different approaches and perspectives on the *U*.

We take inspiration from Marr and Poggio’s (1976) popular “levels of analyses” framework to draw connections between various accounts of the same phenomenon. Marr and Poggio’s framework includes three levels at which information processing systems can be characterized: computational, algorithmic, and implementational. Detailed introductions to this framework are accessible elsewhere (Marr & Poggio, 1976), so we only provide an illustrative example. Consider a thermostat in a car engine. What it does and why can be understood on at least three distinct levels. At the computational level, we can state that a thermostat is there to prevent the engine from overheating (the ‘why’), which it achieves by keeping the ambient temperature at the desired level (the ‘what’). At the algorithmic level, we can understand rather abstractly the process of maintaining the desired temperature (the abstract ‘how’): the thermostat repeatedly compares the current temperature (system input) to the desired level and engages the cooling/heating processes (system output) accordingly. Finally, at the implementational level, we can understand how the algorithmic process can be implemented in a physical system (the concrete ‘how’). For example, in a wax thermostatic element, a continuous comparison process is implemented by a sealed wax pellet that rapidly expands when heated, which opens up valves for the cooler to circulate.

While Marr and Poggio’s framework is often used to gain a fuller understanding of a system in question, we use it to retrospectively make sense of various accounts of the *U*. By synthesizing different theoretical accounts, we provide a basis for discussing these accounts on the theoretical and not only empirical level. What distinguishes the present review from similar integrative reviews (Dubey & Griffiths, 2020; Kidd & Hayden, 2015) is that it presents a more detailed survey of a broad range of theories, with the aim to synthesize them. Specifically, we review *formal specifications* of the theories, in addition to their verbal descriptions, with a focus on a relatively narrow phenomenon.

We review the following theories addressing the *U*: Rational Analysis (Dubey & Griffiths, 2020), Optimal Metacognitive Control (Son & Sethi, 2006), Learning Progress (Gottlieb et al., 2013; Kaplan & Oudeyer, 2007a; Schmidhuber, 1991, 2010), Conflict (Berlyne, 1960), Information Gap (Golman & Loewenstein, 2018; Loewenstein, 1994), Region of Proximal Learning (Metcalfe & Kornell, 2005; Metcalfe et al., 2020), and Achievement Motivation (Atkinson, 1957).

Although we characterize each theory as providing computational- or algorithmic-level descriptions, we will refer to them as *normative* and *process* theories, respectively. While there is considerable overlap between the meanings of these terms, we find that “normative” and “process” more readily capture the distinctive features of these classes of theories. Normative theories do not merely specify the computational objective, but also prescribe a norm for how to achieve it in an optimal way, while process theories describe causal psychological mechanisms.

Normative theories originate from a *function-first* (also called “top-down”) theorizing approach (Griffiths et al., 2010), where theorizers first formulate the computational problem a cognitive system faces and then derive an optimal solution to that problem under a set of assumptions constraining the space of solutions. As mentioned earlier, the resulting theories are normative because they prescribe a norm for how an ideal (or at least rational) agent *should* solve the formulated problem. In other words, these theories explain *why* learners should employ strategies that result in the *U*. In the case of normative theories of curiosity, the postulated computational problem is knowledge optimization (for more details, see Section Normative Theories). These theories normatively state that under a specific set of specific assumptions, a learner would behave optimally if they were maximally curious about tasks for which their knowledge was intermediate. Normative theories correspond to the computational level of description in Marr and Poggio’s framework (Marr & Poggio, 1976). Note that although theories may vary in detail regarding the exact shape of the *U*, they converge on a qualitative pattern in which intermediate levels of knowledge (measured in a variety of ways) are associated with higher curiosity relative to very deficient or complete states of knowledge.

*Process* theories come from an *emergentist* (also called “bottom-up”) approach (McClelland et al., 2010), in which researchers start theorizing by first committing to some low-level mechanistic principles underlying high-level behaviors. A prominent example of the emergentist approach is connectionism, in which researchers test and discuss the implications of parallel and distributed computations in various tasks. Although process theories of curiosity do not constrain their mechanisms to the same extent, they aim to illustrate *how* curiosity arises from a hypothesized causal process involving specific psychological variables and their interactions (for more details, see Section Process Theories). The *U* emerges as a natural consequence of these mechanisms. Process theories posit mental representations and computations involved in the causal chain between inputs (e.g., stimulus properties) and outputs (e.g., information-seeking behavior). These theories correspond to the algorithmic level of description in Marr and Poggio’s framework (Marr & Poggio, 1976).

Our emphasis on the dichotomy between normative and process theories serves both descriptive and instrumental purposes. On the one hand, the dichotomy explicitly characterizes how different theories were constructed. On the other hand, it provides guidance regarding whether and/or how any given pair of theories should be compared. Normative theories are more appropriate for rationalistic epistemological analyses, as they are based on logic and mathematics. Process theories should be ultimately evaluated against the data because they contain propositions about the state of the natural world. A comparison between normative and process theories with respect to the common theoretical subject can be misplaced if they are assumed to be mutually competitive. At the same time, such a comparison can be illuminating if normative and process theories are seen as complementary accounts (Norris & Cutler, 2021).

**Figure 2.**
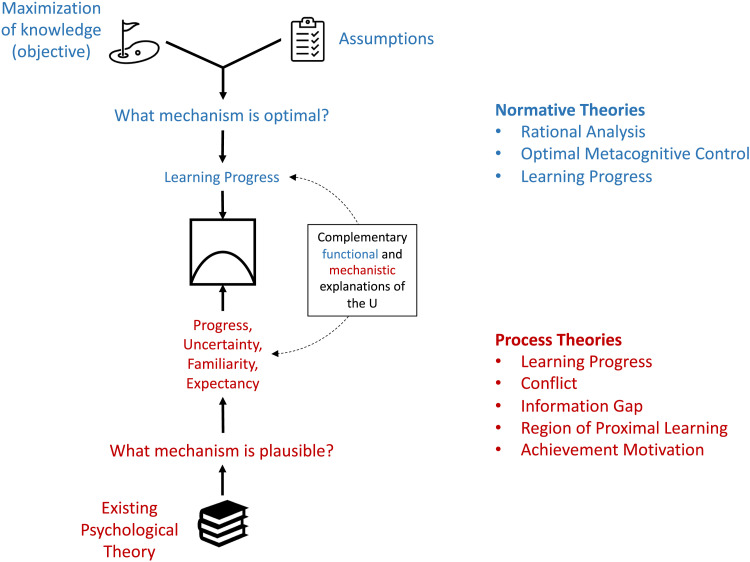
Classification and synthesis of the theories. Theories are classified as either normative (blue) or process (red). Normative theories are formulated by first postulating a computational objective of curiosity and specifying a set of assumptions that constrain the space of solution mechanisms. Different normative theories propose similar objectives and conceptually converge on a solution to maximize learning progress. Under specific assumptions, progress maximization implies the *U*. Process theories incorporate insights from the existing literature and formulate plausible mechanistic accounts of the target phenomenon (the *U*). Different process theories propose mechanisms distinct in the explanatory causal factors involved (progress, uncertainty, familiarity, and expectancy). Normative and process theories supply complementary functional and mechanistic accounts of the *U*.

Figure 2 presents a schematic summary of the relationships between the theories included in this review. These are explained in detail in the following sections. Normative theories fit into a single theoretical framework because they posit a common objective of knowledge maximization for learners and suggest that the *U* can be an optimal solution toward this objective. Conversely, process theories propose distinct mechanisms to explain curiosity, which can be categorized according to the knowledge-related factors involved (uncertainty-, familiarity-, and expectancy-based mechanisms). All these process theories can account for the *U* and thus potentially explain how the normative-theoretic objective can be achieved indirectly, e.g., by an agent lacking the means to compute the optimal solution. Critically, information-seeking behavior suggested by process theories may implicitly achieve the effective maximization of knowledge. In other words, process theories suggest how the normative-theoretic objective can be realized in practice. Thus, knowledge maximization is a common principle underlying both normative and process theories.

The close correspondence between normative and process theories renders the *U* as an *epiphenomenon* reflecting the workings of underlying mechanisms, rather than the description of the mechanism itself. In other words, while it is possible to conceive a mechanism that explicitly represents the system’s knowledge about various tasks and targets those that are in the intermediate range, our theoretical survey compels us to consider another possibility: the tendency to pursue intermediate knowledge tasks emerges as a result of knowledge maximization. Moreover, as explained further, knowledge maximization itself can be (at least in part) an emergent effect of mechanisms that do not explicitly represent knowledge gains.

## NORMATIVE THEORIES

A normative theory proposes a well-motivated objective that poses a problem given limited resources. In the case of normative theories of curiosity, the problem arises due to the limited time available for learning a vast set of tasks. If there was unlimited time to spend on learning tasks, the problem of allocation would not arise in the first place. Normative theories also provide well-performing (often optimal) solutions to the identified problems. These can be constrained by assumptions that theorizers choose to incorporate in their investigation. These assumptions can refer to aspects of the environment or the architecture of the cognitive system, and, thus, constrain the set of possible solutions. We consider three theories of curiosity as normative: Rational Analysis (Dubey & Griffiths, 2020), Optimal Metacognitive Control (Son & Sethi, 2006), and Learning Progress (Kaplan & Oudeyer, 2007a). Although there are differences in formulation, these theories are similar in postulating the same principal objective of curiosity – knowledge optimization. This objective is motivated by the assumption that the adaptive value of knowledge derives from the agent’s ability to achieve its goals better across various situations in a nonstationary environment (see Singh et al., 2010). Critically, despite the apparent differences in formulation, normative theories exhibit striking similarities in mathematical form and commonly indicate the *U* as an optimal solution under specific but relevant conditions. On the one hand, this similarity may seem unexpected, given their largely independent development in different corners of cognitive science and the “top-down” progression of their construction: from the proposed computational problems to idealized mechanisms. Even their formal expressions may seem deceptively distinct (as is the case with the Learning Progress theory). In practice, though, theorizers are likely to tacitly account for the wealth of empirical knowledge when proposing these top-down theories. Whatever the case, no clear contrasts between these theories have been drawn before. Such contrasts are valuable for identifying shared theoretical gaps and taking coherent steps forward.

### Problem(s) of Curiosity

As described above, normative theories are grounded in postulated computational problems that the system in question is assumed to address. More specifically, normative theories of curiosity address the problem of allocating limited time to learning a vast set of tasks. For instance, in Rational Analysis (Dubey & Griffiths, 2020), the postulated problem is to determine how to maximize the *value of knowledge V*, defined as the expected confidence in responding appropriately to each of the *N* stimuli in an agent’s environment. Formally,

$$V=\sum_{i}^{N}p_{i}c_{i}$$ (1)

where *p*_*i*_ is the probability of encountering stimulus *i* in the future and *c*_*i*_ is the agent’s confidence in being able to make correct responses upon encountering stimulus *i*. Exposure time is assumed to increase confidence through a monotonic function, *c*_*i*_ = *f*_*c*_(*t*_*i*_), called the confidence function. The value of knowledge is high when the agent is confident that it can respond appropriately to various stimuli, especially highly expected ones. By maximizing *V*, the agent can maximize long-term rewards.

Another normative theory, the Optimal Metacognitive Control theory (Son & Sethi, 2006), formulates the problem slightly differently. The authors considered how students could maximize their total performance score on a test involving multiple tasks. Formally, the objective of the student is to maximize the total score *S*, defined as the weighted average of competences^1^ across *N* tasks:

$$S=\sum_{i}^{N}w_{i}b_{i}$$ (2)

where *b*_*i*_ is the competence score for task *i*, and *w*_*i*_ is a weighting factor that reflects the importance of task *i* to the student. Competence is also assumed to be a monotonic function of learning time *t*_*i*_; that is, *b*_*i*_ = *f*_*b*_(*t*_*i*_). Son and Sethi referred to this functional relationship between time allocation and competence as the uptake function or, equivalently, the learning curve. Note that this formulation is isomorphic to Dubey and Griffiths’s. In both theories, the learning curve functions are assumed to be monotonic functions of exposure or learning time *t*_*i*_. If the importance of a topic *w*_*i*_ is assumed to reflect its future relevance (i.e., its probability of coming up in a test), the two problem definitions become identical.

Finally, Learning Progress theory identifies the central problem of curiosity as exploring and efficiently learning a large and diverse set of skills (Gottlieb et al., 2013). It is argued that large and diverse skill sets are evolutionarily adaptive, particularly in changing environments (Baldassarre & Mirolli, 2013; Singh et al., 2010). Here, we follow the formal framework introduced by Lopes and Oudeyer (2012; also see Schmidhuber, 2010, for a very similar framework). In this framework, the agent continuously monitors the outcomes of a dynamic multidimensional input *x*^2^. Furthermore, suppose that these outcomes are governed by a hidden function f*(*x*). The agent maintains an estimate of f* as an internal model $\hat{f}\left(x\right)$. As the agent explores its environment and accumulates data *D* = {*o*_1_ = {*x*_1_, f*(*x*_1_)}, …}, it repeatedly updates its internal model to fit the data. Lopes and Oudeyer considered a problem of choosing a new observation *o*′ that minimizes the following goodness-of-fit function, *G*,

$$G\left(D\cup o^{\prime }\right)=-\int_{x}\delta \left[f*\left(x\right),\hat{f}\left(x;D\cup o^{\prime }\right)\right]dx$$ (3)

where *δ* computes the distance between f* and $\hat{f}$ (note that ∫*_x_* denotes the integration over the ambient space of *x*, e.g., ℝ^dims(*x*)^, which comprises the domain for f*). This formulation assumes that the agent wishes to align the output of its internal model with reality. The integral signifies that the total goodness of the model is the combination of “goodnesses” across all possible inputs (see Lopes & Oudeyer, 2012, for details). While this formulation is rather general, a more intuitive, concrete example might help to illustrate the essence of this objective. The function *δ* can be as simple as an absolute prediction error – the absolute difference between the data-informed prediction $\hat{f}\left(x;D\cup o^{\prime }\right)$ and the veridical observation f*(*x*). To simplify further, one could assume one-dimensional observations, which should make it clear that *G* is an index of how well *hatf* predicts the observed outputs of f*.

The similarity of this objective function to those in Equations 2 and 1 is not immediately obvious but can be perceived by observing several details. First, goodness is explicitly a function of *data*. The dependence of knowledge on data is tacit but present in Equations 2 and 1. Formally, *V* and *S* depend on the competence functions (*f*_*c*_ and *f*_*b*_, respectively) that map the task-specific exposure time *t*_*i*_ to competence. Naturally, exposure to a task entails some kind of data assimilation and processing, which result in increased competence. Hence, to make this dependence explicit, one could imagine *V* and *S* as *V*(*D*) and *S*(*D*), respectively, which would require redefining the corresponding competence functions as dependent on the data rather than exposure. Lopes and Oudeyer’s problem definition can be viewed as one of the ways to express competence as a function of data: proximity to the target function (i.e., $\delta [f*(x),\hat{f}(x;D)]$). Thus, all three formulations assume that the objective is optimized by collecting data.

Second, each problem formulation quantifies the quality of knowledge as a composite, or *global*, score, which consists of a combination of *local*, stimulus- or task-specific competences. In the case of *V* and *S*, the combination is a linear sum of competences across discrete situations (i.e., stimuli, or tasks). In contrast, *G* combines competences over different values of continuously represented inputs (the discrete task-set scenario is a special case; see Supplementary Information, Section 3.3). However, the abstract meaning of each objective function is the same; they represent the combined competence of the agent’s knowledge across the set of all possible situations. Section 3.3 of Supplementary Information provides additional details and a formal demonstration of the similarity of the objective functions.

Although local competence functions appear to differ across theories, they represent similar concepts. In Dubey and Griffith’s framework, competence *c*_*i*_ represents an agent’s subjective confidence in obtaining a reward upon encountering a stimulus. The nature of this reward is left open-ended; thus, it can correspond to any arbitrary event, such as goal-state achievement or accurate prediction. Similarly, the notion of task competence (*b*_*i*_), as used in Son and Sethi’s framework, can represent prediction accuracy or goal achievement, depending on the nature of the test. This task competence polymorphism is also present in Lopes and Oudeyer’s framework, although the objective is defined in terms of distance *δ*(⋅, ⋅) ∈ [0, ∞) rather than competence ∈ [0, 1]. For instance, if f*(*x*) represents true outcomes and $\hat{f}\left(x;D\right)$ represents predictions, then a monotonic transformation of $\delta (f*(x),\hat{f}(x))$ (e.g., *e*^−*δ*(⋅, ⋅)^ ∈ (0, 1]) can track the competence of the prediction and correspond to the agent’s subjective confidence in a prediction task. Similarly, if f*(*x*) represents a target state and $\hat{f}\left(x;D\right)$ encodes the actual state achieved by the current model; the distance between the two can indicate the level of competence of the model in a goal achievement task.

It should be noted that despite the high similarities across the three objective functions, they are not completely identical. For example, while *p*_*i*_ in Dubey and Griffith’s model represents the subjective probability of encountering a stimulus in the future, Son and Sethi’s *w*_*i*_ represents a more general (albeit cryptic) notion of subjective importance, which may or may not be a function of stimulus probability. Moreover, the goodness-of-fit objective proposed by Lopes and Oudeyer lacks a weighting component altogether. We view these details, as well as other assumptions (e.g., different competence functions) that each line of work explores, as free parameters of the same underlying system. Different authors investigate the implications of particular parameter values to reach their conclusions, but none seem to commit to empirically testable statements about whether these assumptions are actually valid. As we have seen, these assumptions are not inconsequential to the nature of the resulting objective function and, thus, the optimal solution to the time allocation problem. That is, an optimal agent pursuing the maximization of Equation 1 (where competence is scaled by the probability of a task occurring) would end up with a skill set different from an identical agent in the same environment optimizing Equation 2 (where competence is scaled by the subjective importance of a task).

The similarities between the three normative theories are remarkable, given their apparently independent development. Across the board, the computational problem of curiosity is proposed to be to maximize the general usefulness of knowledge, whether it is formalized as the expected value of knowledge, as in Dubey and Griffiths (2020); or the importance-weighted sum of competence scores, as in Son and Sethi (2006); or the integral of the goodness-of-fit function across all possible inputs (and thus, tasks), as in Lopes and Oudeyer (2012). Abstractly, all theories postulate a knowledge maximization objective that poses the problem of how to rationally choose from a heap of potential situations for learning when it is impossible to learn all of them (e.g., because there is not enough time or energy resources to learn them all).

### Optimal Solutions

Normative theories propose idealistic solutions for problems of curiosity. According to the Rational Analysis theory (Dubey & Griffiths, 2020), the first-order derivative of the objective function *V* with respect to exposure represents the extent to which the expected value of knowledge improves with exposure. It follows that at any given time, it is optimal to be curious about the stimulus for which additional exposure *t*_*i*_ would maximally increase the expected value of knowledge, that is, the stimulus for which the following quantity is maximal,

$$\frac{dV}{{dt}_{i}}=\frac{d\sum_{j}^{N}p_{j}c_{j}}{{dt}_{i}}$$ (4)

Because only one *c*_*j*_ is a function of exposure to stimulus *i*, the derivative can be reduced to

$$p_{i}\frac{{dc}_{i}}{{dt}_{i}}$$ (5)

where $\frac{{dc}_{i}}{{dt}_{i}}$ is interpreted as a local learning gain whose contribution to *V* scales with the probability of that stimulus *p*_*i*_.

Dubey and Griffiths showed that the gain in the expected value of knowledge can be expressed in terms of confidence if two assumptions are maintained. First, past exposure to stimulus *t*_*i*_ is correlated with its future probability of occurrence *p*_*i*_. Second, confidence is exponentially related to exposure (i.e., *c*_*i*_ = *f*_*c*_(*t*_*i*_) = 1 −*e*^−*α_i_t_i_*^, where *α_i_* is the learning rate parameter). With these assumptions, the derivative can be expressed as follows:

$$\frac{dV}{{dt}_{i}}\propto -\log\left(1-c_{i}\right)\times \left(1-c_{i}\right),$$ (6)

which has inverted-*U* shape (see Dubey & Griffiths, 2020, for derivation). Note that these assumptions are sufficient, but not necessary, for producing the *U*^3^. Thus, the optimal strategy for knowledge maximization suggested by the Rational Analysis coincides with the strategy of selecting a stimulus for which confidence is intermediate.

A similar solution was suggested by Son and Sethi (2006); the optimal time allocation depends on the derivative of the objective function at the current level of exposure. The authors explored the implications of different learning curves *f*_*b*_(*t*_*i*_) under the simplifying assumption that the importance weights are constant; that is, *w*_*i*_ = *K* for all *i*. Setting the weight constant makes them independent of *t*_*i*_, implying that the derivative of *S* with respect to *t*_*i*_ can be reduced to the derivative of the learning curve for task *i*. Thus, the optimal time allocation depends solely on the shapes of the learning curve functions of each task and the current exposure levels. In the context of exponential decay learning curves (also used in Dubey & Griffiths, 2020), an optimal agent selects tasks with the lowest level of exposure (i.e., the agent should seek the most novel stimuli). However, if the learning curve is S-shaped (e.g., *b*_*i*_ = *f*_*b*_(*t*_*i*_) = (1 +*e*^−*α_i_t_i_*^)^−1^, where *α_i_* is the learning rate), the derivative that can be expressed as a function of competence (see Supplementary Information, Section 3.3) is

$$\frac{dS}{{dt}_{i}}=\frac{{db}_{i}}{{dt}_{i}}\propto \left(1-b_{i}\right)b_{i}$$ (7)

This function peaks at an intermediate level of competence, resulting in the *U*. In other words, to maximize knowledge of S-shaped tasks, learners can pursue tasks of intermediate competence.

It is important to note that although Dubey and Griffiths (2020) and Son and Sethi (2006) identified different conditions under which optimality implies the *U*, there is no disagreement between the two theories. Son and Sethi argued that the *U* is optimal given S-shaped learning curves (*b*_*i*_ = (1 +*e*^−*α_i_t_i_*^)^−1^) and constant and equal weighting factors (*w*_*i*_ = *K*). Although Dubey and Griffith did not identify this condition, their model actually makes the same prediction. On the other hand, Dubey and Griffiths demonstrated that when weights *p*_*i*_ (or *w*_*i*_) and past exposure *t*_*i*_ are related, the *U* is optimal under the exponential learning curve. Again, while Son and Sethi did not discuss this condition, but this prediction is consistent with their model.

Finally, the solution proposed by Lopes and Oudeyer (2012) is also based on the idea that an agent’s choice should maximally increase the objective function. Because *G* is a function of data, the authors considered using learning progress (*LP*) as a heuristic to the analytical derivative of *G*. They defined it as:

$$LP\left(\left\{x^{\prime }\right\};D\right)=G\left(D\cup \left\{x^{\prime },f*\left(x^{\prime }\right)\right\}\right)-G\left(D\right)$$ (8)

Thus, *LP* quantifies the difference between the total goodness of fit with and without the new data point *x*′. Conceptually, *LP* is similar to the derivatives *dV*/*dt*_*i*_ and *dS*/*dt*_*i*_; however, it expresses how the objective function changes with a discrete change in the dataset rather than with an infinitesimal change in continuous exposure. Lopes and Oudeyer argued that the strategy of greedily sampling the data point that maximizes *LP* is at least quasi-optimal under certain assumptions regarding *G* (specifically, if *G* is assumed to be submodular, i.e., monotonic with diminishing returns). They also demonstrated that a stochastic version of this strategy works well under fewer assumptions regarding *G* (Lopes & Oudeyer, 2012).

According to Dubey and Griffiths (2020), the optimal mechanism implied by Learning Progress theory elicits a preference toward learnable situations that are not too easy or too difficult. They argue that because of this property, Learning Progress theory implies that stimuli of intermediate complexity (always) induce the highest levels of curiosity. However, the formal model from the Learning Progress theory predicts the *U* only under certain assumptions, similar to the Rational Analysis and Optimal Metacognitive Control theories. Specifically, whether the *U* is optimal depends on how local competence functions respond to new data (i.e., what the learning curves are). For example, if a new data point {*x*′} increases task competence the most when the existing dataset *D* is neither too limited nor too saturated, then maximizing *LP* results in the *U*. This is similar to how both Rational Analysis and Optimal Metacognitive Control theories imply the optimality of the *U* if the learning curves are S-shaped. If task competence has a negative exponential shape, *LP*, then curiosity will tend to be greater for more novel tasks unless curiosity also depends on the experience-based weighting factor of each task, as pointed out by Dubey and Griffiths (2020). Therefore, all three formulations make the same predictions regarding when the *U* is optimal. Indeed, from the perspective of Lopes and Oudeyer, the optimal derivative-based solutions provided by Dubey and Griffiths (2020) and Son and Sethi (2006) are analytical analogs of *LP* maximization.

In summary, we posit that the three normative theories not only postulate similar objectives, but also arrive at a similar solution – one that requires giving preference to tasks that provide the greatest gain in an aggregate competence function however it is parameterized. We abstractly refer to this gain in global competence as learning progress and recognize it as a unifying construct that supports conceptual links between all theories in this review. It should be noted that the term has been used widely but somewhat inconsistently in the literature, which might be due to the occasional negligence of the specific objective function that progress is supposed to maximize. The meaning of learning progress rests heavily on the assumptions about the objective function. For example, in Dubey and Griffiths’ (2020) formulation, learning progress leads to the acquisition of useful skill sets (due to task-probability scaling), while in Son and Sethi’s (2006) model, it results in subjectively important skill sets (due to subjective-importance scaling). If tasks are weighted equally, as considered in Lopes and Oudeyer (2012), learning progress is expected to result in the broadest set of skills, as it maximizes the speed of aggregate competence growth, disregarding other aspects of tasks.

### Challenges and Limitations

Because it is common to derive optimal solutions with minimal computational constraints, normative mechanisms are expected to deviate from implementational reality (Chater & Oaksford, 2000; Griffiths et al., 2015). Each normative theory reviewed above suggests computational heuristics that resolve some challenges entailed by optimality.

We have already mentioned Dubey and Griffith’s (2020) Rational Analysis and Son and Sethi’s (2006), Optimal Metacognitive Control suggest that optimal curiosity can be computed from local competence rather than exposure time. This is useful because it allows the agent to behave optimally without explicitly representing and analytically differentiating the learning curves. Although competence-based strategies can be useful in approaching an optimal solution, they are prone to the noisy TV problem (see Kaplan & Oudeyer, 2007b). This is because if, for whatever reason, competence stays intermediate without improving or deteriorating, the agent will become stuck in a task that does not improve its knowledge. For example, if the task was to predict the color of a display, an agent motivated by intermediate competence would become stuck on a “noisy TV” switching randomly between black and white screens. Learning Progress theory proposes a mechanism driven not by intermediate competence per se, but by differences in competence over time. By following the progress in competence over time, the agent can avoid these stationary competence situations.

However, the *LP*-based strategy introduces its own computational challenges. These challenges arise from the difficulty of computing derivatives from finite differences in noisy competence estimates. Any finite-difference computation depends on the “step-size” parameter that dictates the points to be compared. In Equation 8, the step size is implicitly equal to 1 because it corresponds to the number of new data points on which the updated model (i.e., *G*(*D* ∪ {*x*′})) is compared with the old one (*G*(*D*)). Theoretically, smaller step sizes are preferred (for smooth functions) because they are closer to the limit of a finite difference. However, because human performance is noisy, its evaluation can be unreliable for approximating the optimal solution to objective maximization. There seems to be a tradeoff: if the step size is small, the resulting progress estimates can be especially noisy and misleading; the bigger the step size becomes, the more impractical the estimation of the derivative becomes because it requires more data and time.

The three normative theories also share a common caveat. They all consider rational decisions in the context of a known set of tasks or stimuli. However, an important question in curiosity-driven learning is how to gain knowledge about new tasks and stimuli that are outside the set of known tasks (see Etcheverry et al., 2020; Péré et al., 2018). In the real world, individuals are able to not only adopt existing tasks and traverse their mapped out competence trajectories, but also create their own tasks representations (goals and progress measures). While an active area of research in artificial intelligence (see Colas et al., 2020; Gaven et al., 2025), this so-called *autotelic* aspect of learning is lacking from the current normative theories that focus on selecting a given set of tasks.

Asking how curious agents come to discover new tasks would require an extension of the existing normative theories. In this new setting, there is still the problem of efficient allocation of limited resources to learning known tasks, but also additional problems of novel task discovery and dealing with potential reassessments of known tasks (e.g., if new information suddenly expands the space of hypotheses and makes the agent realize that there were aspects of the familiar task it did not consider). Whether discovered or reassessed, these novel tasks change the computational target (e.g., the value of knowledge), and an optimal agent would need to somehow account for that. Moreover, task discovery and task reassessment can be viewed as tasks themselves, which implies that they compete with the other (learning) tasks for limited resources. However, improving at the task of novel task discovery could decrease rather than increase the expected value of knowledge in the currently formulated normative frameworks, as the agent could be incompetent at novel tasks. Simultaneously, introducing novel tasks has a potential of extending the absolute expected value of knowledge indefinitely if the agent can learn them reliably. Such considerations call for a re-evaluation of normative theories of curiosity because they all adopt a simplifying assumption of a known and fixed task space.

## PROCESS THEORIES

The narrative (and often development) of normative theory begins with the identification of a computational objective characterized by certain assumptions about the environment in which the computational system is embedded. In the normative theories reviewed earlier, the *U* is implied by the optimization of the objective under specific constraints. In contrast, *process theories* begin by targeting a phenomenon and constructing a plausible mechanism to explain it in light of existing theoretical knowledge. Thus, while normative theories explain *why* the *U* might emerge, process theories focus on *how* the *U* does emerge.

In the following sections, we review four additional process theories: Conflict theory (Berlyne, 1960), Information Gap theory (Golman & Loewenstein, 2018; Loewenstein, 1994), Region of proximal learning theory (Metcalfe & Kornell, 2005; Metcalfe et al., 2020), and Achievement Motivation theory (Atkinson, 1957). We also discuss a part of the Learning Progress theory that proposes a mechanistic account of human curiosity (Kaplan & Oudeyer, 2007a, 2007b)^4^. We identify four distinct mechanistic proposals within these theoretical frameworks. We call these progress-, uncertainty-, familiarity-, and expectancy-based mechanisms. We speculate that not only are these mechanisms compatible with progress computation, but they may also play unique and complementary roles in optimizing knowledge.

It is important to realize that, although these mechanisms were developed through a non-normative approach, they could be ultimately regarded as the mechanisms to effectively increase knowledge, thus “approximate” normative behavior. Therefore, the *U* predicted by the process theories can also be understood from the normative knowledge-maximization perspective. Thus, despite following different paths of theoretical development, both normative and process theories eventually lead to the same conclusion that the *U* arises due to the knowledge-maximization principle of curiosity. We clarify this point by explaining each theory.

### Progress

Optimal solutions for reaching the postulated objectives can be regarded as possible yet unfeasible mechanisms^5^. The Learning Progress theory happens to propose a computationally cheap mechanism that approximates optimality via numerical differentiation. Indeed, curiosity based on learning progress was initially proposed as a pragmatic mechanism for efficient autonomous learning in artificial agents (Oudeyer et al., 2007; Schmidhuber, 1991), and only later demonstrated to exhibit optimality under specific assumptions (Lopes & Oudeyer, 2012). Consequently, Kaplan and Oudeyer (2007a, 2007b) formulated the *Learning Progress Hypothesis* proposing that the human brain hosts a mechanism that computes progress (i.e., the numerical derivative of competence) to modulate motivation. This can be expressed as

$$C_{i}\propto {LP}_{i}$$ (9)

where *C*_*i*_ denotes curiosity regarding task *i* and *LP*_*i*_ (not exactly the same as *LP* from Equation 8) is the numerically estimated rate of change in competence. Conceptually, this kind of mechanism is the most direct approximation of the ideal solutions proposed by normative theories, which makes progress-based mechanisms especially suited for knowledge maximization. However, the Learning Progress theory does not provide a canonical concrete mechanistic specification of *LP*, and currently, there are multiple algorithmically distinct mechanisms that one could construe as progress-based (e.g., Oudeyer & Kaplan, 2007; Poli et al., 2020; Poli et al., 2022; Schmidhuber, 1991; Ten, Gottlieb, et al., 2021; Ten, Kaushik, et al., 2021).

As discussed earlier, progress-based mechanisms do not always predict the *U*; The *U* is expected under a specific set of circumstances. For example, in a single-task scenario with an S-shaped learning trajectory, a progress-maximizing agent would be maximally curious in the middle of the learning process when learning gains per unit of time are relatively high. Similarly, progress-based mechanisms would also predict a general preference for intermediately difficult tasks if all available tasks had the same learning curve. Then, tasks on which competence is intermediate would always be preferred to those on which competence is low (and very slowly rising) or those on which competences is high (and does not rise anymore).

While progress-based mechanisms are powerful and can account for the *U*, existing implementations rely on the retrospective measurement of progress. Even algorithms that can infer progress for novel situations work by extrapolating the previously observed progress from similar situations (Baranes & Oudeyer, 2013; Poli et al., 2022). Thus, progress-based mechanisms require explicit (numeric or analytical) differentiation of previous records of task competence, even if they aim to guess the future. In contrast, the mechanisms described further may provide means for signaling potential progress without tracking and comparing task competence over time.

### Uncertainty

Perhaps because of their historical precedence, uncertainty-based mechanisms are among the most prominent process models of curiosity. These mechanisms drive the agent to seek information whenever a situation produces “important” subjective uncertainty among potential responses. Two theories feature an uncertainty-based mechanism: conflict theory (Berlyne, 1960) and Information Gap theory (Golman & Loewenstein, 2018; Loewenstein, 1994). Conflict theory explains the *U* by describing the process of *conflict arousal* (Berlyne, 1957) in which external stimulation primes a set of incompatible behavioral responses such as motor responses (e.g., pressing one of the buttons to complete a task), perceptual responses (e.g., recognizing a colleague at work based on appearance), epistemic responses (e.g., thinking up an answer to a trivia question), and predictions (for example, anticipating an outcome of a sports game). Curiosity is assumed to be positively related to conflict, which, in turn, is the product of two factors:

$$C_{i}\propto \bar{E}\left(R_{i}\right)\cdot H\left(R_{i}\right)$$ (10)

where *R*_*i*_ is a set of activation strengths of various response tendencies prompted by stimulus *i*; $\bar{E}\left(\cdot \right)$ is the average strength (or energy) of the responses which represents the “importance” or the scale of uncertainty; *H* is Shannon’s entropy function: *H*(*R*_*i*_) = ∑_*r*_*j*_∈*R*_*i*__ *p*(*r*_*j*_) log*p*(*r*_*i*_), which reflects the uncertainty over activated responses *r*_*j*_ in *R*_*i*_, with *p* mapping *r*_*j*_ to probability (i.e., relative strength of the response). The strength of response activation depends not only on external stimulation and the agent’s current goal(s) but also on existing knowledge (e.g., in connectionist systems, patterns of activation depend on connection weights that represent knowledge). As noted by Berlyne (1957), Information Theory, as used here, applies to situations in which the activated responses are mutually incompatible, and thus conflicting. States of conflict are assumed to be biologically significant and cause an organism to seek information^6^.

How does this mechanistic formulation yield the *U*? The *U* pattern arises because the quantities $\bar{E}\left(\cdot \right)$ and *H*(⋅) are both related to knowledge and have antagonistic effects on conflict. In a person with insufficient knowledge, a stimulus is assumed to only weakly activate the associated responses, resulting in high uncertainty but very small average activation. The resulting conflict (i.e., the right-hand side of 10) and curiosity are small in this situation. Conversely, a knowledgeable person might react to the same stimulus with only one but strongly activated response. In this case, the average activation is relatively high, but the uncertainty is close to zero, leading to low conflict and curiosity. Curiosity is predicted to be relatively high at a level of knowledge that produces several strongly activated competing responses. For example, if asked, “what is the hottest planet in the Solar System?”, a person with partial knowledge could consider two possibilities, e.g., “was it Venus or Mercury?” and thus be uncertain about two highly activated responses.

Information Gap theory (Golman & Loewenstein, 2018) proposes a mechanism similar to Berlyne’s conflict arousal. Here, curiosity is characterized as an information-seeking drive for maximizing the subjective utility of one’s cognitive state. The implied mechanism predicts curiosity to be higher for more uncertain and salient beliefs:

$$C_{i}\propto w_{i}\cdot H\left(\pi_{i}\right)$$ (11)

where *H*(π*_i_*) stands for the uncertainty about a particular belief *i*, and *w*_*i*_ is a scalar attention (or importance) weight associated with the belief^7^. The resemblance to Berlyne’s conflict model (Equation 10) is clear. In fact, the manner in which the two models give rise to the *U* is analogous. For example, we can assume that knowledge affects different aspects of cognitive state in opposite directions. New knowledge can reduce the uncertainty of a belief, while increasing its attentional weight. Indeed, Golman and Loewenstein (2018) suggested two mechanisms of cognitive attention: importance-based and surprise-based modulation, both of which can increase with new knowledge. Thus, when the knowledge underlying a belief is insufficient, curiosity is low because of a lack of attention (and despite high uncertainty). When knowledge is complete, curiosity is also low, owing to the absence of uncertainty (and despite the possibly high attention)^8^.

Importantly, these (weighted) uncertainty-based mechanisms could serve as an approximate, though likely imperfect, solution for the knowledge optimization objective postulated by normative theories. Low curiosity in states of low uncertainty directs individuals away from situations in which learning has stagnated. Simultaneously, low curiosity in states of low importance (e.g., when response potentials or attention-weights are low) safeguards learners from situations where learning is possible in principle but unlikely due to the insufficiency of the current knowledge. When both uncertainty and importance are intermediate, on the other hand, information-seeking behavior is likely to lead to effective learning. Thus, uncertainty-based mechanisms should drive agents away from situations where learning is improbable and towards situations where learning is likely, resulting in knowledge maximization.

### Familiarity

A different type of curiosity mechanism based on situational familiarity was proposed by Metcalfe et al. in the proximal learning theory^9^ (Metcalfe et al., 2020). In this mechanism, the processing of epistemic queries, such as trivia questions is accompanied by a metacognitive process that assesses how close one is to retrieving an answer. Being “on the verge” of finding an answer is being in the *region of proximal learning* – a state characterized by increased motivation and engagement, i.e., curiosity. One important assumption is that while some states of knowledge may not be sufficient for successful answer retrieval, they might be enough to conjure a metacognitive feeling of familiarity and motivate further information-seeking.

Although the theory is not formally specified in mathematical notation, the authors suggest a certain shape for the relationship between curiosity and knowledge. Specifically, curiosity is proposed to convexly increase the metacognitive feeling of knowing the target of the query, but only up to the point at which the target can be successfully retrieved. We formally express this proposal as follows:

$$C_{i}\propto \left(1-{AR}_{i}\right)\cdot g\left(F_{i}\right)$$ (12)

where *i* is the index of a query, *g* is a nonlinear convex function of *F*_*i*_, is the metacognitive judgment of knowing the answer to the query, and *AR*_*i*_ is a binary variable representing whether answer retrieval was successful (*AR*_*i*_ = 1) or failed (*AR*_*i*_ = 0). In this formulation, curiosity is a function of familiarity and not uncertainty.

Although the precise functional relationship between knowledge and *F*_*i*_ and *AR*_*i*_ is not specified, it is natural to view these variables as either directly dependent on knowledge. As the knowledge abound a query accumulates, *g*(*F*_*i*_) increases until the answer is known (i.e., retrievable), at which point the product of the two factors discontinuously becomes zero. The convexity of *g* indicates that curiosity increases with a sense of knowledge. This does not affect the *U* itself (i.e., regardless of whether the functional form, the *U* holds); however, it deserves attention. The authors proposed *tip-of-the-tongue* states to support this convexity intuition. Tip-of-the-tongue states are characterized by a particularly intense urge to seek information when a cue activates a large amount of irrelevant information about the target, thereby increasing *F*_*i*_, but fails to elicit the target itself (Metcalfe et al., 2020).

The tip-of-the-tongue phenomenon also illustrates the distinction between familiarity and uncertainty. A tip-of-the-tongue state is not a state of conflict, as it involves activation of mutually compatible responses. Thus, the absence of response conflict does not necessarily indicate a complete lack of relevant experience or knowledge. In other words, conflict may be a sufficient factor for curiosity, but not a necessary one. This makes familiarity-based curiosity suitable for the early stages of learning, signifying situations with prior experience in the absence of strongly activated competing response tendencies. At the same time, uncertainty and familiarity overlap – conflict cannot occur if learners are not familiar with potential responses. An interesting follow-up question is whether familiarity- and uncertainty-based factors have independent (additive) effects on curiosity, or whether they interact as two aspects of a single process. For example, familiarity could be defined as average activation strength, or the importance weight could be defined as an increasing function of familiarity. This proposal merges familiarity- and uncertainty-based accounts.

Similarly to the uncertainty-based mechanisms, familiarity-based curiosity could also approximate the ideal solution towards the maximization of knowledge. The mechanism in Equation 12 predicts curiosity to peak at the highest level of familiarity induced by a query before the target information can be retrieved. The inability to retrieve a response indicates the opportunity for learning, and higher levels of familiarity indicate higher potential for successful encoding and retention of information. When familiarity is low, on the other hand, processing and retention of new information is assumed to be less effective (see Witherby & Carpenter, 2022). When a satisfactory response is accessible (*AR* = 1) there is nothing new to learn. Thus, curiosity based on contextual familiarity with an uncertain situation is likely to lead to effective knowledge accumulation.

### Expectancy

Atkinson’s (1957) achievement-motivation theory features another mechanism to explain the *U*. Originally, the theory was developed to explain motivation in goal pursuit; however, we briefly explain its relevance to curiosity-driven behavior. This mechanism is distinct from uncertainty- and familiarity-based mechanisms in two ways. First, it is based on a metacognitive evaluation of goal achievement *expectancy*, rather than uncertainty or familiarity. Second, it was implicated in the decision-making context of choosing among alternative goals rather than stimuli such as trivia questions.

Achievement motivation is conceptualized as a particular parameterization of the general formulation of motivation (see Supplementary Information, Section 3.3). Namely, assuming a positive achievement motive and preference for subjectively difficult goals, Atkinson expressed motivation, such as curiosity, as a function of expectancy:

$$C_{i}\propto P_{i}\cdot \left(1-P_{i}\right),$$ (13)

where the expectancy term *P*_*i*_ is defined as the subjective probability of succeeding on task *i*. This function implies that intermediate expectancies are preferred. Note a striking similarity to Equation 7, suggesting the Atkinson’s mechanism is optimal under specific assumptions. Achievement Motivation theory was originally proposed to explain variation in goal values, but we consider it useful to relate the theory to curiosity. Self-challenge tendencies predicted by the Achievement Motivation theory direct individuals toward risky goals, but also away from goals that appear unequivocally impossible in the current state of one’s subjective competence (which reflects knowledge). Pursuing risky goals entails attempting various actions and observing their effects. In computational reinforcement learning, action-observation pairs are often used as data for learning intelligent action policies. In fact, competence-based intrinsic motivation has been widely recognized as a form of curiosity-driven exploration (Oudeyer & Kaplan, 2007; Schmidhuber, 2010) in autotelic agents that learn by selecting and pursuing (and sometimes inventing) their own goals (Colas et al., 2020). Thus, pursuing risky goals can be viewed as a form of information seeking (see Gottlieb & Oudeyer, 2018).

Not only does the expectancy-based mechanism seem compatible with other mechanisms involving uncertainty and familiarity, but it might also be complementary to and dependent on them in the context of lifelong learning. Consider a situation in which an individual decides whether to pursue learning a complex skill such as playing piano. While evaluating the equipotentiality of the response tendencies activated by thinking about piano playing (i.e., conflict) is unfeasible in making this decision, the evaluation of expectancy can be helpful. For instance, for knowledge maximization purposes, it may be beneficial for an individual to forgo learning the piano if eventual mastery seems genuinely impossible. It could also be beneficial to consider acquiring other skills if playing the piano has already been mastered. The different roles of uncertainty- and expectancy-based mechanisms across different active learning situations make them complementary in the overall lifelong learning process. Moreover, the computation of expectancy itself could be partially based on the metacognitive representation of familiarity; for example, one could be more optimistic about learning to play the piano if one had seen it being played before. In fact, expectancy is closely related to the concept of self-efficacy, which depends not only on vicarious experiences (e.g., seeing someone perform a skill), but also on personal performance, verbal persuasion, and emotional arousal (Bandura, 1977).

The expectancy-based mechanism has direct relevance to progress maximization. It enables us to estimate expected progress before ever trying anything like the activity in consideration. No progress should be expected from novel activities with very high expectancy because these activities are deemed already mastered. Similarly, little progress can be expected from activities with minimal expectancy because such activities may not provide any positive feedback for learning. Most learning should be expected from intermediate-expectancy activities. Thus, the expectancy-based mechanism can also contribute to knowledge maximization.

### Summary of Process Theories

In summary, we reviewed four instances of process theories, each of which explains curiosity based on different core factors (i.e., progress, uncertainty, familiarity, and expectancy). Despite the different formulations, the mechanisms in all these theories can explain the *U*. Furthermore, weighted uncertainty, retrieval-gated familiarity, and expectancy-weighted achievement incentive can all be viewed as markers of learning opportunities. We speculate that following these markers (approximately) maximizes knowledge, which is supported by various studies reporting positive effects of curiosity on memory (Fastrich et al., 2018; Gruber et al., 2014; Kang et al., 2009; Wade & Kidd, 2019; Witherby & Carpenter, 2022). Thus, process theories could provide alternative implementation-level accounts of mechanisms that subserve knowledge maximization. The fact that process theories seem to be generally aligned with normative constraints suggests that the proposed curiosity mechanisms are *rational* – i.e., they elevate learning and exploration as high as possible given the constraints imposed by the environment and our cognitive architecture.

## DISCUSSION

Theories of curiosity have been burgeoning. However, these theories have been developed in different contexts and fields and are often viewed as distinct, leaving little discussion on how they are related to each other. Here, we synthesized several prominent curiosity theories by leveraging the fact that they all address the inverted-*U* shaped relationship between knowledge and curiosity (Figure 2; Silvia, 2006). Specifically, we proposed that the principle of knowledge maximization serves as a common link that binds these theories together. Normative theories unanimously propose that the goal of curiosity is to address the problem of optimal allocation of limited time in the service of knowledge maximization. Process theories, which historically precede normative theories, propose distinct but compatible mechanistic descriptions that are also in line with the normative computational objective. Our synthesis invites a perspective in which the *U* emerges as an epiphenomenon to mechanisms that work toward knowledge maximization.

### Main Takeaways

We have demonstrated that knowledge maximization is the guiding principle that integrates different theories of the *U*. In addition, we highlight two main takeaways. First, although normative theories provide an explanation for the *U* and predict it as an optimal solution in certain computational settings, none of these theories insist on the general optimality of the *U*. This means that the effectiveness of pursuing an intermediate level of knowledge (*k*) *per se* is not absolute, but depends on whether particular assumptions about the environment and various features of the learning trajectories of different tasks hold. For example, as pointed out by Dubey and Griffiths (2020), if stimuli have a uniform probability of occurrence, and knowledge is an exponential function of stimulus exposure, then the preference for stimuli with intermediate confidence would be suboptimal. In addition, even in the conditions we identified as optimal by sampling intermediate-confidence stimuli (e.g., when the learning curve is *S*-shaped), optimality actually depends on other stimuli among which people make a choice. Therefore, even if we believe that humans are approximately optimal in task selection, we should not expect to observe the *U* everywhere. Although it is important for the mechanistic theory of curiosity to explain the *U* (Silvia, 2006), it is just as important to explain whether and how a curiosity mechanism optimizes the value of knowledge (or deviates from optimality) in a given setting.

This flexibility of normative theories highlights a significant advantage over bottom-up theorization. Normative theorization allows the theory to get ahead of experimental data and sometimes propose novel experimental settings to capture different aspects of a more fundamental mechanism that operates beyond a particular effect, such as the *U*.

Second, our observation of the compatibility among different implementation-level mechanisms fits well with a “pluralist” view of curiosity as a multifaceted motivational state arising from several distinct mechanisms (Modirshanechi et al., 2023; Poli et al., 2024). How these mechanisms operate simultaneously remains an important question for future research. One perspective is that information seeking at any given point in time is pulled in the direction of multiple intrinsic (and extrinsic) rewards (Modirshanechi et al., 2023; Poli et al., 2024; Sharot & Sunstein, 2020; see Juechems & Summerfield, 2019). Another (compatible) possibility is that different mechanisms play distinct roles in the same multipart learning process (Metcalfe & Kornell, 2005; Murayama et al., 2019; Poli et al., 2024; Schwartenbeck et al., 2019; Xu et al., 2020), implying either different computational objectives or different approaches to meet the same objective under different sets of constraints. We encourage researchers to consider such hybrid mechanisms, and hope that the current review (of algorithmic implementations) will serve as a common ground for clearly contrasting newly proposed mechanisms with existing theories.

The explicit pluralistic account of curiosity is supported by the influential I/D-curiosity framework (Litman, 2005, 2019; Litman & Jimerson, 2004). The framework proposes two types of curiosity that determine the motivational and affective profile of exploratory behavior in different circumstances. Curiosity as a feeling of Interest (I-type) compels individuals to seek situations with higher levels of uncertainty (e.g., browsing for a new series on a streaming platform), while curiosity as a feeling of Deprivation (D-type) induces an uncomfortable drive-like state that individuals want to resolve by seeking information (e.g., watching the last episode to see how the story ends; FitzGibbon et al., 2020). Put differently, with I-type curiosity one wants to approach uncertainty, while with D-type, one wants to get away from it. In a study conducted by Litman et al. (2005), the self-reported I-type curiosity trait predicted the intensity of state curiosity for trivia questions to which the participants reported not knowing the answer. The I-type curiosity trait did not reliably predict curiosity for questions for which participants reported a tip-of-the-tongue (TOT) state (i.e., an intense feeling of knowing the answer coinciding with the failure to retrieve it). On the other hand, the D-type curiosity trait predicted the level of state curiosity about the TOT questions, but not “don’t know” questions. These findings resonate with our previously expressed idea about the complementarity between uncertainty-based and expectancy-based mechanisms. Specifically, uncertainty-based mechanisms might underlie the D-type curiosity, which arise in response to subjective uncertainty, while expectancy-based mechanisms might underlie the I-type curiosity, which can arise in the absence of subjective uncertainty.

Accepting these connections implies a few interesting predictions that can be tested empirically. The I/D framework would imply that uncertainty-based vs. expectancy-based motivational states have different affective profiles, distinct neural mechanisms, and different levels of motivational intensity (Litman, 2005, 2019). These insights could be of interest to computational theories of curiosity, as they offer a potential solution (which is distinct from a learning-progress mechanism) to the noisy TV problem associated with competence-based mechanisms, like the expectancy-based mechanism. While I-type curiosity can cause the agent to approach a potentially unsolvable task, the more intense negative emotions associated with D-type curious states may lead to the eventual disengagement out of frustration. Another implication of the parallels between process theories and the I/D framework suggests that both I-type and D-type curiosity can have a U-shaped relationship with knowledge. Interestingly, Atkinson’s original model (Atkinson, 1957) contains an extra degree of freedom (see Overview of Atkinson’s Achievement Motivation Framework in Supplementary Information) allowing for a reversed pattern between motivation and expectancy. Such variability in the relationship between motivation and expectancy is consistent with a recently proposed reward-learning framework of interest development (Murayama et al., 2019), which holds that the tendency to experience I-type motivation is gradually shaped by the (intrinsic) reward history of an individual. By this account, the willingness to challenge oneself, and thus, the optimality of one’s motivation with respect to knowledge maximization, is not a given, but depends on the experienced outcomes of uncertain situations.

### Related Work

An emerging body of literature studies curiosity within the framework of knowledge graphs (also called semantic networks). For example, Bassett et al. (Bassett & Sporns, 2017; Lydon-Staley et al., 2021; Patankar et al., 2022) and Murayama et al. (Donnellan et al., 2022; Murayama, 2022) provide different accounts of the gap formation process in growing knowledge graphs. We note that the “knowledge gaps” described in this knowledge network literature are conceptually and formally distinct from the information gaps discussed in Loewenstein’s theory (1994). The information gap in Loewenstein’s and Berlyne’s work is defined as the Shannon entropy of the probability distribution of the predicted responses or answers (Equations 10, 11). In the knowledge network literature, knowledge gaps are formalized as topological cavities (Patankar et al., 2022) or missing edges (Murayama, 2022). While these different notions of “knowledge gaps” and “information gaps” seem to be related conceptually, their functional and/or algorithmic correspondence is unclear.

The models of curiosity discussed in this review are related to a recent computational model of aesthetic valuation by Brielmann and Dayan (2022). In this model, the aesthetic value of a perceptual stimulus depends on two factors that vary differently with exposure: the immediate sensory predictability of the current stimulus and the change in the expected future predictability of other stimuli. Because of their opposing effects on aesthetic valuation, continued exposure to an unusual stimulus is predicted to first increase and then eventually decrease the aesthetic value, resulting in a characteristic *U*-shaped pattern. Although Brielmann and Dayan’s model is not intended to explain curiosity, it may tap into the same computational problem as knowledge optimization. Namely, the preference for aesthetically pleasing stimulation could reflect the adaptive function of a motivational mechanism that pushes agents to expand knowledge by exploring atypical stimuli but simultaneously prevents knowledge from overfitting to short-term sporadic trends in the environment.

Another phenomenon that is closely related to curiosity is boredom. Like curiosity, boredom is an affective state that depends, at least partly, on knowledge. What may be boring for a knowledgeable person may be exciting for an ignorant person, and vice versa. In fact, boredom has been predicted (and demonstrated) to have a *U*-shaped relationship with task difficulty, such that tasks that either exceed or fall short of one’s available cognitive resources are more likely to induce boredom (Westgate & Wilson, 2018). Moreover, an aversive state of boredom encourages individuals to pursue more mentally challenging states, such as interest or curiosity. This hints at yet another complementary (though not specialized) mechanism for knowledge maintenance, although it is not specialized. Under- or over-stimulation causing boredom could signal the overqualification or insufficiency of knowledge in the current task, so the unpleasantness of such states could be an effective way to eventually direct individuals toward learning progress.

### Limitations and Future Directions

We have purposefully limited the scope of the current review to discuss the theories concerning the *U* in sufficient detail. However, the theoretical landscape of curiosity research extends beyond the relationship between curiosity and knowledge. For example, several authors have proposed qualitative distinctions between different kinds of curiosity, such as diversive vs. specific curiosity, perceptual vs. epistemic curiosity (Berlyne, 1966), curiosity as a feeling of interest vs. vs curiosity as a feeling of deprivation (FitzGibbon et al., 2020; Litman, 2005; Shin & Kim, 2019). A comprehensive unified theory of curiosity should explain whether and how normative considerations (i.e., what is curiosity is for?) predict different kinds of curiosity. Moreover, researchers have identified different motives underlying non-instrumental information seeking that are both separate from motives related to knowledge maximization and can potentially compete with epistemically optimal behavior (e.g., Brändle et al., 2023; Kobayashi et al., 2019; Niehoff & Oosterwijk, 2020; Raab et al., 2022; Sharot & Sunstein, 2020). Reconciling these findings with the knowledge maximization principle would also be valuable for future research on intrinsically motivated information-seeking.

## Acknowledgments

We would like to thank Aditya Singh for numerous insightful discussions around various themes throughout the manuscript. We also thank the Self-Regulation Hub at the Hector Research Institute for helpful feedback and discussion.

## Funding Information

This research was supported by the Alexander von Humboldt Foundation (the Alexander von Humboldt Professorship endowed by the German Federal Ministry of Education and Research) to Kou Murayama, the Japan Society for the Promotion of Science awarded to Michiko Sakaki (22H00088), and the ANR Grant awarded to Pierre-Yves Oudeyer (ANR-19-CHIA-0004).

## Author Contributions

A.T.: Conceptualization; Writing – original draft; Writing – review & editing. P.Y.O.: Writing – review & editing. M.S.: Conceptualization; Writing – review & editing. K.M.: Conceptualization; Writing – review & editing.

## Notes

^1^ The notion of competence, although a more objective index of ability, is similar to confidence in the previous framework. While it would be optimal for students to base their decisions on objective measures of competence, such measures are generally unavailable; thus, decision-makers have to resort to subjective evaluations of competence, that is, confidence.^2^ For concreteness, one could think of inputs and their outcomes as temporally adjacent internal states. Thus, the agent continuously infers the next state from the current state (see Oudeyer & Kaplan, 2007).^3^ Dubey and Griffiths actually noted that the *U* arises for any monotonic function between exposure and confidence (p. 459). However, as we clarify below, this is not entirely correct. Their conclusion partly depends on the shape of the relationship between exposure and confidence.^4^ Note that our usage of the term “theory” is rather loose in this instance. We view the Learning Progress theory as a set of propositions. Some of these propositions pertain to the optimization of the objective function and thus serve as a basis for normative claims. Others stem from the assumed cognitive limitations (e.g., limitations in the abilities to estimate and represent learning curves) and maintain descriptions of the proposed process.^5^ We elaborate on the potentially confusing duality of process and normative theories in Section 3.3 of Supplementary Information.^6^ Berlyne initially assumed that conflict helped organisms avoid the aversive state of high arousal by driving them toward optimal levels of stimulation (Berlyne, 1960; Madsen, 1981). However, he later seems to have relaxed the reliance on arousal and arousal potential as explanatory causal variables for curiosity (Silvia, 2006). With these revisions, the causal pathway from conflict to curiosity does not involve arousal.^7^ Note that Golman and Loewenstein (2018) did not explicitly formulate curiosity as such. This conclusion is based on our understanding of their theory. We explain our rationale in more detail in Supplementary Information, Section 3.3.^8^ However, we note that the apparent similarity between conflict theory and Information Gap theory does not go beyond the involvement of scaled entropy in curiosity. In Golman and Loewenstein’s (2018) framework, curiosity toward a belief depends not only on uncertainty but also on a non-cognitive belief-valence factor. Moreover, the scaling factor *w*_*i*_ is more multifaceted than the average response activation parameter $\bar{E}\left(\cdot \right)$ in Berlyne’s model.^9^ The theory is more comprehensive than how we represent it here and involves multiple metacognitive mechanisms (including those analogous to uncertainty-based mechanisms). Here, we highlight the familiarity-based process because it is distinct and proposed exclusively by the Region of Proximal Learning theory.

## Supplementary Material



## References

[bib1] Atkinson, J. W. (1957). Motivational determinants of risk-taking behavior. Psychological Review, 64(6), 359–372. 10.1037/h0043445, 13505972

[bib2] Baldassarre, G., & Mirolli, M., Eds. (2013). Intrinsically motivated learning in natural and artificial systems. Berlin Heidelberg: Springer-Verlag. 10.1007/978-3-642-32375-1

[bib3] Bandura, A. (1977). Self-efficacy: Toward a unifying theory of behavioral change. Psychological Review, 84(2), 191–215. 10.1037/0033-295X.84.2.191, 847061

[bib4] Baranes, A., & Oudeyer, P.-Y. (2013). Active learning of inverse models with intrinsically motivated goal exploration in robots. Robotics and Autonomous Systems, 61(1), 49–73. 10.1016/j.robot.2012.05.008

[bib5] Baranes, A., Oudeyer, P.-Y., & Gottlieb, J. (2015). Eye movements reveal epistemic curiosity in human observers. Vision Research, 117, 81–90. 10.1016/j.visres.2015.10.009, 26518743

[bib6] Bassett, D. S., & Sporns, O. (2017). Network neuroscience. Nature Neuroscience, 20(3), 353–364. 10.1038/nn.4502, 28230844 PMC5485642

[bib8] Berlyne, D. E. (1957). Uncertainty and conflict: A point of contact between information-theory and behavior-theory concepts. Psychological Review, 64(6), 329–339. 10.1037/h0041135, 13505970

[bib9] Berlyne, D. E. (1960). Conflict, arousal, and curiosity. New York, NY, US: McGraw-Hill Book Company. 10.1037/11164-000

[bib7] Berlyne, D. E. (1966). Curiosity and exploration. Science, 153(3731), 25–33. 10.1126/science.153.3731.25 5328120

[bib10] Brändle, F., Stocks, L. J., Tenenbaum, J. B., Gershman, S. J., & Schulz, E. (2023). Empowerment contributes to exploration behaviour in a creative video game. Nature Human Behaviour, 7(9), 1481–1489. 10.1038/s41562-023-01661-2, 37488401

[bib11] Brielmann, A. A., & Dayan, P. (2022). A computational model of aesthetic value. Psychological Review, 129(6), 1319–1337. 10.1037/rev0000337, 35786988

[bib12] Chater, N., & Oaksford, M. (2000). The rational analysis of mind and behavior. Synthese, 122(1), 93–131. 10.1023/A:1005272027245

[bib13] Colas, C., Karch, T., Sigaud, O., & Oudeyer, P.-Y. (2020). Autotelic agents with intrinsically motivated goal-conditioned reinforcement learning: A short survey. arXiv. 10.48550/arXiv.2012.09830

[bib14] Donnellan, E., Sakaki, M., & Murayama, K. (2022). From curiosity to interest: Accumulated knowledge supports long-term persistence of information-seeking behavior. In I. Cogliati Dezza, E. Schulz, & C. M. Wu (Eds.), The drive for knowledge (pp. 31–52). Cambridge University Press. 10.1017/9781009026949.003

[bib15] Dubey, R., & Griffiths, T. L. (2020). Reconciling novelty and complexity through a rational analysis of curiosity. Psychological Review, 127(3), 455–476. 10.1037/rev0000175, 31868394

[bib16] Etcheverry, M., Moulin-Frier, C., & Oudeyer, P.-Y. (2020). Hierarchically organized latent modules for exploratory search in morphogenetic systems. Advances in Neural Information Processing Systems, 33, 4846–4859.

[bib17] Fastrich, G. M., Kerr, T., Castel, A. D., & Murayama, K. (2018). The role of interest in memory for trivia questions: An investigation with a large-scale database. Motivation Science, 4(3), 227–250. 10.1037/mot0000087, 30221181 PMC6133257

[bib18] FitzGibbon, L., Lau, J. K. L., & Murayama, K. (2020). The seductive lure of curiosity: Information as a motivationally salient reward. Current Opinion in Behavioral Sciences, 35, 21–27. 10.1016/j.cobeha.2020.05.014

[bib19] Gaven, L., Carta, T., Romac, C., Colas, C., Lamprier, S., Sigaud, O., & Oudeyer, P.-Y. (2025). MAGELLAN: Metacognitive predictions of learning progress guide autotelic LLM agents in large goal spaces. arXiv. 10.48550/arXiv.2502.07709

[bib20] Geana, A., Wilson, R. C., Daw, N., & Cohen, J. D. (2016). Boredom, information-seeking, and exploration. In Proceedings of the 38th Annual Conference of the Cognitive Science Society. Austin, TX: Cognitive Science Society.

[bib21] Golman, R., & Loewenstein, G. (2018). Information gaps: A theory of preferences regarding the presence and absence of information. Decision, 5(3), 143–164. 10.1037/dec0000068

[bib22] Gottlieb, J., & Oudeyer, P.-Y. (2018). Towards a neuroscience of active sampling and curiosity. Nature Reviews Neuroscience, 19(12), 758–770. 10.1038/s41583-018-0078-0, 30397322

[bib23] Gottlieb, J., Oudeyer, P.-Y., Lopes, M., & Baranes, A. (2013). Information-seeking, curiosity, and attention: Computational and neural mechanisms. Trends in Cognitive Sciences, 17(11), 585–593. 10.1016/j.tics.2013.09.001, 24126129 PMC4193662

[bib24] Griffiths, T. L., Chater, N., Kemp, C., Perfors, A., & Tenenbaum, J. B. (2010). Probabilistic models of cognition: Exploring representations and inductive biases. Trends in Cognitive Sciences, 14(8), 357–364. 10.1016/j.tics.2010.05.004, 20576465

[bib25] Griffiths, T. L., Lieder, F., & Goodman, N. D. (2015). Rational use of cognitive resources: Levels of analysis between the computational and the algorithmic. Topics in Cognitive Science, 7(2), 217–229. 10.1111/tops.12142, 25898807

[bib26] Gruber, M. J., Gelman, B. D., & Ranganath, C. (2014). States of curiosity modulate hippocampus-dependent learning via the dopaminergic circuit. Neuron, 84(2), 486–496. 10.1016/j.neuron.2014.08.060, 25284006 PMC4252494

[bib27] Juechems, K., & Summerfield, C. (2019). Where does value come from? Trends in Cognitive Sciences, 23(10), 836–850. 10.1016/j.tics.2019.07.012, 31494042

[bib28] Kang, M. J., Hsu, M., Krajbich, I. M., Loewenstein, G., McClure, S. M., Wang, J. T., & Camerer, C. F. (2009). The wick in the candle of learning: Epistemic curiosity activates reward circuitry and enhances memory. Psychological Science, 20(8), 963–973. 10.1111/j.1467-9280.2009.02402.x, 19619181

[bib29] Kaplan, F., & Oudeyer, P.-Y. (2007a). In search of the neural circuits of intrinsic motivation. Frontiers in Neuroscience, 1(1), 225–236. 10.3389/neuro.01.1.1.017.2007, 18982131 PMC2518057

[bib30] Kaplan, F., & Oudeyer, P.-Y. (2007b). The progress-drive hypothesis: An interpretation of early imitation. In C. L. Nehaniv & K. Dautenhahn (Eds.), *Imitation and Social Learning in Robots, Humans and Animals* (pp. 361–378). Cambridge University Press. 10.1017/CBO9780511489808.024

[bib31] Kidd, C., & Hayden, B. Y. (2015). The psychology and neuroscience of curiosity. Neuron, 88(3), 449–460. 10.1016/j.neuron.2015.09.010, 26539887 PMC4635443

[bib32] Kidd, C., Piantadosi, S. T., & Aslin, R. N. (2012). The goldilocks effect: Human infants allocate attention to visual sequences that are neither too simple nor too complex. PLoS ONE, 7(5), e36399. 10.1371/journal.pone.0036399, 22649492 PMC3359326

[bib33] Kobayashi, K., Ravaioli, S., Baranés, A., Woodford, M., & Gottlieb, J. (2019). Diverse motives for human curiosity. Nature Human Behaviour, 3(6), 587–595. 10.1038/s41562-019-0589-3, 30988479

[bib34] Litman, J. (2005). Curiosity and the pleasures of learning: Wanting and liking new information. Cognition and Emotion, 19(6), 793–814. 10.1080/02699930541000101

[bib35] Litman, J. (2019). Curiosity: Nature, dimensionality, and determinants. In K. A. Renninger & S. E. Hidi (Eds.), *The Cambridge handbook of motivation and learning* (pp. 418–442). Cambridge University Press. 10.1017/9781316823279.019

[bib36] Litman, J., Hutchins, T., & Russon, R. (2005). Epistemic curiosity, feeling-of-knowing, and exploratory behaviour. Cognition & Emotion, 19(4), 559–582. 10.1080/02699930441000427

[bib37] Litman, J. A., & Jimerson, T. L. (2004). The measurement of curiosity as a feeling of deprivation. Journal of Personality Assessment, 82(2), 147–157. 10.1207/s15327752jpa8202_3, 15041521

[bib38] Loewenstein, G. (1994). The psychology of curiosity: A review and reinterpretation. Psychological Bulletin, 116(1), 75–98. 10.1037/0033-2909.116.1.75

[bib39] Lopes, M., & Oudeyer, P.-Y. (2012). The strategic student approach for life-long exploration and learning. *2012 IEEE International Conference on Development and Learning and Epigenetic Robotics (ICDL)*, 1–8. 10.1109/DevLrn.2012.6400807

[bib40] Lydon-Staley, D. M., Zhou, D., Blevins, A. S., Zurn, P., & Bassett, D. S. (2021). Hunters, busybodies and the knowledge network building associated with deprivation curiosity. Nature Human Behaviour, 5(3), 327–336. 10.1038/s41562-020-00985-7, 33257879 PMC8082236

[bib41] Lyew, T., Ikhlas, A., Sayed, F., Vincent, A., & Lydon-Staley, D. M. (2023). Curiosity, surprise, and the recall of tobacco-related health information in adolescents. Journal of Health Communication, 28(7), 446–457. 10.1080/10810730.2023.2224254, 37318238 PMC10330854

[bib42] Madsen, K. B. (1981). Berlyne’s theory. In H. I. Day (Ed.), Advances in intrinsic motivation and aesthetics (pp. 19–38). Boston, MA: Springer US. 10.1007/978-1-4613-3195-7_2

[bib43] Marr, D., & Poggio, T. A. (1976). From understanding computation to understanding neural circuitry. A.I. Memo 357.

[bib44] McClelland, J. L., Botvinick, M. M., Noelle, D. C., Plaut, D. C., Rogers, T. T., Seidenberg, M. S., & Smith, L. B. (2010). Letting structure emerge: Connectionist and dynamical systems approaches to cognition. Trends in Cognitive Sciences, 14(8), 348–356. 10.1016/j.tics.2010.06.002, 20598626 PMC3056446

[bib45] Metcalfe, J., & Kornell, N. (2005). A Region of Proximal Learning model of study time allocation. Journal of Memory and Language, 52(4), 463–477. 10.1016/j.jml.2004.12.001

[bib46] Metcalfe, J., Schwartz, B. L., & Eich, T. S. (2020). Epistemic curiosity and the region of proximal learning. Current Opinion in Behavioral Sciences, 35, 40–47. 10.1016/j.cobeha.2020.06.007, 33709011 PMC7943031

[bib47] Modirshanechi, A., Kondrakiewicz, K., Gerstner, W., & Haesler, S. (2023). Curiosity-driven exploration: Foundations in neuroscience and computational modeling. Trends in Neurosciences, 46(12), 1054–1066. 10.1016/j.tins.2023.10.002, 37925342

[bib48] Murayama, K. (2022). A reward-learning framework of knowledge acquisition: An integrated account of curiosity, interest, and intrinsic–extrinsic rewards. Psychological Review, 129(1), 175–198. 10.1037/rev0000349, 35099213

[bib49] Murayama, K., FitzGibbon, L., & Sakaki, M. (2019). Process account of curiosity and interest: A reward-learning perspective. Educational Psychology Review, 31, 875–895. 10.1007/s10648-019-09499-9

[bib50] Niehoff, E., & Oosterwijk, S. (2020). To know, to feel, to share? Exploring the motives that drive curiosity for negative content. Current Opinion in Behavioral Sciences, 35, 56–61. 10.1016/j.cobeha.2020.07.012

[bib51] Norris, D., & Cutler, A. (2021). More why, less how: What we need from models of cognition. Cognition, 213, 104688. 10.1016/j.cognition.2021.104688, 33775402 PMC8346944

[bib53] Oudeyer, P.-Y., & Kaplan, F. (2007). What is intrinsic motivation? A typology of computational approaches. Frontiers in Neurorobotics, 1, 6. 10.3389/neuro.12.006.2007, 18958277 PMC2533589

[bib52] Oudeyer, P.-Y., Kaplan, F., & Hafner, V. V. (2007). Intrinsic motivation systems for autonomous mental development. IEEE Transactions on Evolutionary Computation, 11(2), 265–286. 10.1109/TEVC.2006.890271

[bib54] Patankar, S. P., Zhou, D., Lynn, C. W., Kim, J. Z., Ouellet, M., Ju, H., Zurn, P., Lydon-Staley, D. M., & Bassett, D. S. (2022). Curiosity as filling, compressing, and reconfiguring knowledge networks. arXiv. 10.48550/arXiv.2204.01182

[bib55] Péré, A., Forestier, S., Sigaud, O., & Oudeyer, P.-Y. (2018). Unsupervised learning of goal spaces for intrinsically motivated goal exploration. arXiv. 10.48550/arXiv.1803.00781

[bib57] Poli, F., Meyer, M., Mars, R. B., & Hunnius, S. (2022). Contributions of expected learning progress and perceptual novelty to curiosity-driven exploration. Cognition, 225, 105119. 10.1016/j.cognition.2022.105119, 35421742 PMC9194910

[bib58] Poli, F., O’Reilly, J. X., Mars, R. B., & Hunnius, S. (2024). Curiosity and the dynamics of optimal exploration. Trends in Cognitive Sciences, 28(5), 441–453. 10.1016/j.tics.2024.02.001, 38413257

[bib56] Poli, F., Serino, G., Mars, R. B., & Hunnius, S. (2020). Infants tailor their attention to maximize learning. Science Advances, 6(39), eabb5053. 10.1126/sciadv.abb5053, 32967830 PMC7531891

[bib59] Raab, H. A., Foord, C., Ligneul, R., & Hartley, C. A. (2022). Developmental shifts in computations used to detect environmental controllability. PLoS Computational Biology, 18(6), e1010120. 10.1371/journal.pcbi.1010120, 35648788 PMC9191713

[bib60] Schmidhuber, J. (1991). Curious model-building control systems. [Proceedings] 1991 IEEE International Joint Conference on Neural Networks, 2, 1458–1463. 10.1109/IJCNN.1991.170605

[bib61] Schmidhuber, J. (2010). Formal theory of creativity, fun, and intrinsic motivation (1990–2010). IEEE Transactions on Autonomous Mental Development, 2(3), 230–247. 10.1109/TAMD.2010.2056368

[bib62] Schwartenbeck, P., Passecker, J., Hauser, T. U., FitzGerald, T. H., Kronbichler, M., & Friston, K. J. (2019). Computational mechanisms of curiosity and goal-directed exploration. eLife, 8, e41703. 10.7554/eLife.41703, 31074743 PMC6510535

[bib63] Sharot, T., & Sunstein, C. R. (2020). How people decide what they want to know. Nature Human Behaviour, 4(1), 14–19. 10.1038/s41562-019-0793-1, 31932690

[bib64] Shin, D. D., & Kim, S. (2019). Homo curious: Curious or interested? Educational Psychology Review, 31(4), 853–874. 10.1007/s10648-019-09497-x

[bib65] Silvia, P. J. (2006). Exploring the psychology of interest (p. 276). Oxford, New York: Oxford University Press. 10.1093/acprof:oso/9780195158557.001.0001

[bib66] Singh, A., & Manjaly, J. A. (2021). The effect of information gap and uncertainty on curiosity and its resolution. Journal of Cognitive Psychology, 33(4), 403–423. 10.1080/20445911.2021.1908311

[bib67] Singh, S., Lewis, R. L., Barto, A. G., & Sorg, J. (2010). Intrinsically motivated reinforcement learning: An evolutionary perspective. IEEE Transactions on Autonomous Mental Development, 2(2), 70–82. 10.1109/TAMD.2010.2051031

[bib68] Son, L. K., & Sethi, R. (2006). Metacognitive control and optimal learning. Cognitive Science, 30(4), 759–774. 10.1207/s15516709cog0000_74, 21702835

[bib69] Spitzer, M. W. H., Janz, J., Nie, M., & Kiesel, A. (2024). On the interplay of curiosity, confidence, and importance in knowing information. Psychological Research, 88(1), 101–115. 10.1007/s00426-023-01841-9, 37278725 PMC10243256

[bib70] Ten, A., Gottlieb, J., & Oudeyer, P.-Y. (2021). Intrinsic rewards in human curiosity-driven exploration: An empirical study. Proceedings of the Annual Meeting of the Cognitive Science Society, 43.

[bib71] Ten, A., Kaushik, P., Oudeyer, P.-Y., & Gottlieb, J. (2021). Humans monitor learning progress in curiosity-driven exploration. Nature Communications, 12(1), 5972. 10.1038/s41467-021-26196-w, 34645800 PMC8514490

[bib72] Wade, S., & Kidd, C. (2019). The role of prior knowledge and curiosity in learning. Psychonomic Bulletin & Review, 26, 1377–1387. 10.3758/s13423-019-01598-6, 31079309

[bib73] Walker, E. L. (1981). The quest for the inverted U. In H. I. Day (Ed.), Advances in intrinsic motivation and aesthetics (pp. 39–70). Boston, MA: Springer US. 10.1007/978-1-4613-3195-7_3

[bib74] Westgate, E. C., & Wilson, T. D. (2018). Boring thoughts and bored minds: The MAC model of boredom and cognitive engagement. Psychological Review, 125(5), 689–713. 10.1037/rev0000097, 29963873

[bib75] Witherby, A. E., & Carpenter, S. K. (2022). The rich-get-richer effect: Prior knowledge predicts new learning of domain-relevant information. Journal of Experimental Psychology: Learning, Memory, and Cognition, 48(4), 483–498. 10.1037/xlm0000996, 33539165

[bib76] Xu, H. A., Modirshanechi, A., Lehmann, M. P., Gerstner, W., & Herzog, M. H. (2020). Novelty is not Surprise: Human exploratory and adaptive behavior in sequential decision-making. bioRxiv. 10.1101/2020.09.24.311084 PMC8205159 34081705

