# Supplementary material for: The Curious U: Integrating Theories Linking Knowledge and Information-Seeking Behavior
Source: Open Mind (Camb). 2025 Oct 17;9:1763–85. doi: 10.1162/OPMI.a.41 (PMC12618012; doi:10.1162/OPMI.a.41)
Supplement: Supplementary file 1 [file opmi-09-1763-s001.pdf]

The Curious *U*: Integrating Theories Linking Knowledge and Information-Seeking Behavior

Supplementary Information

Alexandr Ten[1], Pierre-Yves Oudeyer[2], Michiko Sakaki[1], and Kou Murayama[1]

[1]Hector Research Institute of Education Sciences and Psychology, University of Tübingen, Tübingen, Germany
[2]Flowers Team, INRIA, University of Bordeaux, Talence, France

October 7, 2025

1. Alternative Form of Objective Function in Lopes & Oudeyer

To determine how the objective function of Lopes and Oudeyer (**lopes˙strategic˙2012**) resembles those of Dubey and Griffiths (**dubey˙reconciling˙2020**) and Son and Sethi (**son˙metacognitive˙2006**), it is important to understand how the functions f^* and $\hat{f}$ (introduced in Section "Problem(s) of Curiosity") are defined. Namely, f^* and $\hat{f}$ are mixture models that combine multiple input-outcome mappings simultaneously. Whether they output, for example, predictions or motor directives for any given x depends on x itself. Formally,

$$f^*(x) = \sum_{i=1}^{N} \beta_i(x) g_i^*(x; \theta_i^*)$$

and

$$\hat{f}(x; D) = \sum_{i=1}^{N} \beta_i(x) \hat{g}_i(x; \hat{\theta}_i, D)$$

where N is the number of tasks; $g_i^*(x; \theta_i^*)$ and $\hat{g}_i(x; \hat{\theta}_i, D)$ are task-specific functions (experts) mapping inputs to task-specific outputs (e.g., predictions, motor directives), given their respective task-specific parameter vectors θ_i^* and $\hat{\theta}_i$ ($\hat{g}_i$ also depends on D); $\beta_i(x)$ is an indicator (mixture) function that maps values from a certain region of x to 1, and values outside this region to 0. The subdomains where these indicator functions are evaluated as 1 are assumed to be non-overlapping[1]. Conceptually, indicator β_i acts as a switch that turns on a single task-i-specific function, depending on the input.

Assuming that δ computes the Euclidean distance (as an example), Equation (3) from the main text can be rewritten as follows:

$$G(D) = -\int_x \| \sum_{i=1}^{N} \beta_i(x) \big(g_i^*(x; \theta_i^*) - \hat{g}_i(x; \hat{\theta}_i, D) \big) \| dx$$

[1] Lopes and Oudeyer (**lopes˙strategic˙2012**) do not strictly impose such constraints on the mixture functions, allowing them to vary freely between 0 and 1 and have overlapping subdomains. However, assuming these constraints is helpful for understanding the similarities between the different formulations of competence across theories.

which clarifies that integrating over the full domain x involves computing the sum of N tasks for the differences between $g^*(x)$ and $\hat{g}(x)$ for each x. Because of the assumption of nonoverlapping subdomains of the indicator functions, we can equivalently write the following:

$$G(D) = -\sum_{i=1}^{N} \int_{x_i} \|\left(g_i^*(x_i;\theta_i^*) - \hat{g}_i(x_i;\hat{\theta}_i, D)\right)\| dx_i$$

where x_i is the subdomain of x where $\beta_i(x)$ is 1. The integral inside the sum computes a measure of the competence of the estimated task-specific function $\hat{g}_i$ as its overall Euclidean proximity to g_i^*. Although this definition of task-specific competence is more detailed than $f_c(t_i) = c_i$ and $f_b(t_i) = b_i$ (from Equations (1) and (2) in the main text, respectively), it expresses a similar notion of knowledge quality with respect to task i.

2. S-Shaped Learning Curves in the Objective Function

Let

$$S = \sum_{i}^{N} w_i b_i$$

where b_i is a sigmoid function σ of t parameterized by α_i

$$b_i = \sigma(t_i) = \left(1 + e^{-\alpha_i \cdot t_i}\right)^{-1}$$

Then, the derivative of S with respect to time spent learning task i is

$$\frac{dS}{dt_t} = \frac{d\sum_i^N w_i \sigma(t_i)}{dt_t}$$

If $w_i = K$ for all $i \in \{1, ..., N\}$, where K is a constant, then

$$\frac{dS}{dt_i} = \frac{dK\sigma(t_i)}{dt_i}$$

As $\frac{dKb_{j\neq i}}{dt_i} = 0$, and given that $\sigma'(\cdot) = (1 - \sigma(\cdot))\sigma(\cdot)$, we can apply the chain rule to obtain

$$\frac{dS}{dt_i} = K(1 - \sigma(t_i))\sigma(t_i)\alpha_i$$
$$\propto (1 - b_i)b_i$$

3. Duality of Normative and Process Theories

The distinction between normative and process theories can be fuzzy. This is because of the rather loose usage of the term ”theory” we have adopted in the main article. We view any collection of propositions that describe how or why a phenomenon occurs as a theory. Thus, an optimal solution derived in a normative theory may provide a valid computational recipe that, if executed, would achieve optimality. At the same time, a process theory can contain normative statements about what agents should do to reach certain objectives.

The Learning Progress theory discussed in Sections “Normative Theories” and “Progress” is particularly ambiguous regarding how it is best categorized, which is why we classify it as both. There is a complete normative aspect of the theory that states what would be optimal for a given objective. And while the solution (equation (4) in the main text) is computationally unfeasible, the theory discusses computable approximations to the ideal.

An example of a process theory that contains elements of a normative theory is Golman and Loewenstein’s Information Gap theory. In this theory, curiosity arises out of a need to optimize the subjective utility function. While this makes it similar to other normative theories discussed in the main text, it falls short from a complete normative account. First, it does not explicitly justify the nature of the objective; for example, why should one try to maximize belief valence and minimize uncertainty? Second, the theory does not propose an optimal decision strategy for maximizing subjective utility beyond suggesting that such a solution would involve evaluating and selecting lotteries on future cognitive states, contingent on the sequences of actions (**golman˙information˙2018**).

4. Overview of Golman and Loewenstein's Information-Gap Framework

The theory's framework revolves around the concept of *cognitive state* (denoted $\mathcal{S}$) – a set comprising N beliefs and a vector of attention weights corresponding to each belief. Beliefs in the cognitive state are assumed to be uncertain; accordingly, they are represented as questions with multiple possible answers characterized by varying degrees of subjective conviction. Formally, each belief can be characterized as a set $\mathcal{B}_i = \{Q_i, \mathcal{A}_i, \pi_i\}$, where Q_i is i-th belief's question (from a set of questions $\mathcal{Q}$, e.g., $\mathcal{Q} = \{Q_1 =$ "What is the hottest planet in the Solar System?", $Q_2 =$ "What is my name?", ..., $Q_N\}$); $\mathcal{A}_i = \{A_{i,1}, A_{i,2}, ...\}$ is a set of *answers* corresponding to Q_i that comprises a sample space for a probability distribution π_i, representing one's subjective convictions regarding a belief. Finally, the attention-weight vector $\boldsymbol{w} = (w_1, ..., w_N)$ indicates how much attention each belief receives at any given point in time, and can be interpreted as a collection of "importance" scores of each belief.

In this framework, beliefs can be characterized by their utility score determined by belief uncertainty and belief valence. Specifically, belief utility increases with valence and decreases with uncertainty. The utility of an entire cognitive state is defined in terms of individual belief utilities. The original utility function of Golman and Loewenstein (**golman˙information˙2018**) is rather complex as it involves material rewards and belief valences in addition to belief attention and uncertainty. To reveal the underlying similarity with Berlyne's conflict theory, we presented a simplified version that ignores material value and assumes constant belief valence.

The utility of a cognitive state is a function of individual convictions ($\boldsymbol{\pi}_i$) and attention ($\boldsymbol{w}$) at any given time,

$$u(\mathcal{S}) = \sum_{i=1}^{N} w_i \Big[K - H(\boldsymbol{\pi}_i) \Big]$$

where K is the constant belief valence and $H(\boldsymbol{\pi}_i)$ is Shannon's entropy of $\boldsymbol{\pi}_i$. Thus, the utility of a cognitive state is the weighted sum of the belief utilities that are positively related to belief valence and negatively related to belief uncertainty. As individuals are assumed to

prefer cognitive states with higher utility, they are driven to minimize the uncertainty of their attended (e.g., important) beliefs. The belief with the highest $w_i \cdot H(\boldsymbol{\pi}_i)$ value would be the belief that can potentially increase u the most (assuming that H can be reduced to 0). Hence, curiosity could be characterized as quantity proportional to $w_i \cdot H(\boldsymbol{\pi}_i)$.

5. Overview of Atkinson's Achievement Motivation Framework

Achievement Motivation theory holds that the tendency to select a goal, which we denote as the value of goal G, depends on three factors:

$$G = M \cdot P \cdot I$$

where M is the motive, P is the expectation of achieving a goal, and I is the incentive value of the achieved goal. Motive (M) refers to the individual's capacity to enjoy certain outcomes. In particular, Achievement Motivation theory concerns the achievement motive, the extent to which an individual derives pleasure from achieving challenging goals. It is helpful to consider motives as personal values of an individual. Thus, the achievement motive indicates the extent to which an individual values achievement. Expectancy (P) corresponds to the subjective assessment of an individual's ability to achieve a goal. It is intuitive to think of expectancy as related to relatively concrete goals (e.g., hitting a strike in bowling, drawing a self-portrait, or solving an equation), but it may also relate to more abstract skills (e.g., being good at bowling, drawing, or math). Finally, incentive value (I) refers to the amount of hedonic value an individual derives from achieving a goal or mastering a skill. It is possible to relate the equation above to previous theories more by considering the abstract functional roles of each component: M can be regarded as a subjective importance weight for the utility contained in accomplishing a challenging task $P \times I$.

To make an inverted-U prediction between expectancy and motivation, Atkinson defined achievement motivation to be a particular parameterization of G. Specifically, he assumed

that the motive is constant (e.g., $M = 1$) and $I = (1 - P)$. The former is used to simplify: we consider only those goals for which the motives are equal. The latter expresses the intuition that achieving challenging goals is incentivizing. Adopting these assumptions reduces the general form above to $G = P \cdot (1 - P)$, as presented in the main text.
